# The Impact of Human Lipoaspirate and Adipose Tissue-Derived Stem Cells Contact Culture on Breast Cancer Cells: Implications in Breast Reconstruction

**DOI:** 10.3390/ijms21239171

**Published:** 2020-12-01

**Authors:** Asim Ejaz, Katherine S. Yang, Kaushik P. Venkatesh, Somaiah Chinnapaka, Lauren E. Kokai, J. Peter Rubin

**Affiliations:** Department of Plastic Surgery, University of Pittsburgh, Pittsburgh, PA 15261, USA; ejaza@upmc.edu (A.E.); ksy6@pitt.edu (K.S.Y.); kpv5@pitt.edu (K.P.V.); chinnapakas@upmc.edu (S.C.); kokail@upmc.edu (L.E.K.)

**Keywords:** adipose-derived stem cells, lipoaspirates, breast cancer cells, cell cycle and proliferation

## Abstract

Background: Autologous fat transfer in the form of lipoaspirates for the reconstruction of the breast after breast cancer surgery is a commonly used procedure in plastic surgery. However, concerns regarding the oncologic risk of nutrient-rich fat tissue are widely debated. Previous studies have primarily focused on studying the interaction between adipose-derived stem cells (ASCs) and breast cancer cells. Methods: In this study, we performed a comprehensive analysis of the paracrine- and contact-based interactions between lipoaspirates, ASCs and breast cancer cell lines. An inverted flask culture method was used to study the contact-based interaction between lipoaspirates and breast cancer cells, while GFP-expressing breast cancer cell lines were generated to study the cell–cell contact interaction with ASCs. Three different human breast cancer cell lines, MCF-7, MDA-MB-231 and BT-474, were studied. We analyzed the impact of these interactions on the proliferation, cell cycle and epithelial-to-mesenchymal (EMT) transition of the breast cancer cells. Results: Our results revealed that both lipoaspirates and ASCs do not increase the proliferation rate of the breast cancer cells either through paracrine- or contact-dependent interactions. We observed that lipoaspirates selectively inhibit the proliferation of MCF-7 cells in contact co-culture, driven by the retinoblastoma (Rb) protein activity mediating cell cycle arrest. Additionally, ASCs inhibited MDA-MB-231 breast cancer cell proliferation in cell–cell contact-dependent interactions. Quantitative real-time PCR revealed no significant increase in the EMT-related genes in breast cancer cells upon co-culture with ASCs. Conclusion: In conclusion, this study provides evidence of the non-oncogenic character of lipoaspirates and supports the safety of clinical fat grafting in breast reconstruction after oncological surgical procedures. In vivo studies in appropriate animal models and long-term post-operative clinical data from patients are essential to reach the final safety recommendations.

## 1. Introduction

The global burden of breast cancer surpasses all other cancers, and is the most common malignancy in females [1]. Advancements in diagnostic and therapeutic approaches have resulted in improved survival rates. However, breast-conserving therapies or mastectomies are associated with disfiguring and stigmatizing outcomes for patients. Autologous fat transfer (AFT), pioneered by Coleman in the late 1990s, is an invaluable tool used routinely by plastic surgeons for tissue reconstruction and augmentation [2,3], and AFT for breast augmentation and reconstruction has become increasingly popular [4].

Fat tissue abundance, as well as the autologous nature and minimal invasiveness of the procedure, are the attractive factors supporting the broader utilization of AFT. In addition to high patient and surgeon satisfaction, AFT is associated with improved scar healing, and reductions in pain and fibrosis following oncological radiation therapy [1,5,6,7,8,9,10,11]. Adipose-derived stem cells (ASCs) residing within the adipose tissue likely play a key role in maintaining graft volume retention and regenerative functions [12]. ASCs contribute to these functions by secreting a plethora of paracrine factors that promote angiogenesis, cell migration and differentiation [2].

Despite the clinical benefits, AFT is associated with a few addressable drawbacks, such as graft resorption and interference with diagnostic tissue imaging. More importantly, the greatest concern associated with AFT is the possibility of cancer-promoting interactions between paracrine-rich adipose tissue and remnant tumor cells in the tumor bed, which can result in locoregional tumor recurrence. This concern has been supported by results from recent studies showing that in vitro expanded ASCs enhance breast cancer cell growth and migration in vitro and in rodent models [13,14,15]. Interactions between ASCs and breast cancer cells increase cytokine production and malignant transformation of the breast cancer cells via adipsin and Src/Sox/miR-302b mediated signaling [14,16]. Further, a recent study employing in vitro differentiated adipocytes showed that fatty acids released through adipocyte lipolysis drive breast cancer cell proliferation and differentiation [17]. Furthermore, breast cancer cells induce morphological and functional changes in ASCs, altering them to function as cancer-associated fibroblasts. Such cells promote tumor progression, angiogenesis and endothelial-to-mesenchymal transition (EMT), a key event involved in tumor metastasis [18]. Similarly, Rowan et al. demonstrated enhanced kidney, lung and spleen metastasis upon the injection of ASCs together with breast cancer cells [19].

However, most studies that suggest ASCs increase tumor progression use co-culture or in vivo models, which is inconsistent with the clinical scenario composed of intact adipose parcels injected into a large tissue bed for breast reconstruction. In addition, most of the studies relied on in vitro differentiated adipocytes or paracrine interactions between cells as experimental models [14,16,17,20,21,22]. On the other hand, a recent study from our lab demonstrated the oncological safety of lipofilling as part of the surgical platform for breast reconstruction after cancer therapy. We had used a clinically relevant animal model and reported no increase in tumor size, proliferation, histological grade, or metastatic spread [23]. These results are supported by the meta-analysis of clinical results, in which a long-term (5 year) follow up study of AFT involving 587 total patients (287 AFT and 300 controls, matched for age, type of oncological surgery, tumor invasiveness, and disease stage) showed no increase in locoregional recurrence of cancer. Rather, a lower but non-significant decrease was observed [1].

This study aimed to provide comprehensive insight into the interactions between breast cancer cells, lipoaspirates and ASCs. To our knowledge, this is the first example of the paracrine and contact co-culturing of breast cancer cells with lipoaspirate or ASCs from the same donors. We analyzed the effects of these interactions on the proliferation of breast cancer cells and analyzed the underlying mechanisms.

## 2. Results

### 2.1. Lipoaspirate Contact Co-Culture Reduces the Proliferation Rate of Breast Cancer Cell Lines

**Figure 1:**
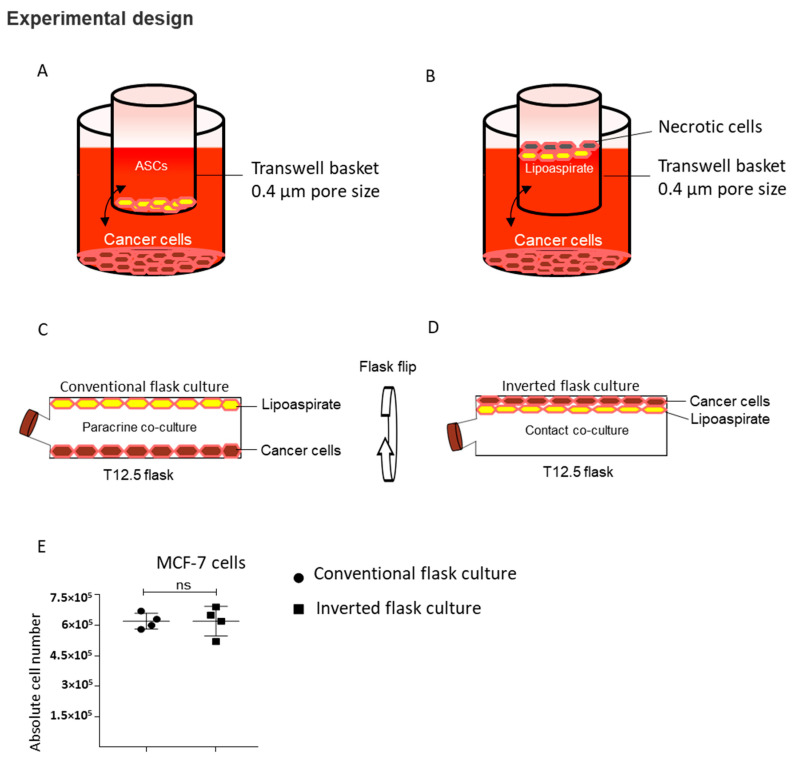
Contact and paracrine co-culture experimental design. (**A**) Paracrine culture setup of cancer cells with adipose-derived stem cells (ASCs) using 6-well transwell plates. (**B**) Paracrine culture setup of cancer cells with lipoaspirates. (**C**,**D**) Modified paracrine (**C**) and contact (**D**) co-culture of cancer cells with lipoaspirate using T12.5 cm^2^ flask. (**E**) Comparison of MCF-7 cells proliferation in conventional and inverted flask culture (*n* = 4). ns = non-significant.

An important concern pertaining to the use of fat grafts for the post-oncological reconstruction of the breast is the possible aggravation of the remnant breast cancer cells upon interaction with transferred autologous fat in the form of lipoaspirates. Although several clinical and animal studies have demonstrated similar or lower incidences of cancer recurrence from post-oncological reconstruction with fat graft compared to other means of breast reconstruction [1,23,24], there still exist substantial data indicating a pro-oncogenic role of lipoaspirates and adipose-derived stem cells [14,16,20,22]. To the best of our knowledge, the most clinically relevant scenario has yet to be studied, i.e., a direct cell–cell contact interaction between lipoaspirates and breast cancer cells. In addition, studies investigating the interaction between adipocytes and breast cancer cells either rely on in vitro differentiated ASCs [14,17,21] or have used transwell or a 6-well co-culture system [14,20]. One drawback of using transwell or a 6-well co-culture system is that the lower density adipocytes float and may be damaged by hypoxia (Figure 1A,B). These damaged adipocytes release inflammatory cytokines that may change the co-culture milieu. To overcome this technical issue and simulate the clinical scenario of breast cancer cells and lipoaspirate interaction, we performed a paracrine and contact co-culture in a closed flask setting, as shown in (Figure 1C,D), respectively.

Utilizing the buoyancy property of lipoaspirate, we performed a conventional culture where, after seeding the breast cancer cells and adding lipoaspirate, we incubated the flask using conventional methods that enable the cancer cells and lipoaspirate to stay apart, thus allowing only the paracrine interaction (Figure 1C). To achieve contact between lipoaspirates and breast cancer cells, we incubated the flasks in an inverted position (Figure 1D) which enabled the floating lipoaspirates to come into contact with breast cancer cells. We observed similar growth kinetics of MCF-7 in our conventional and inverted flask culture settings, supporting the utilization of these culture settings for co-culture studies (Figure 1E).

**Figure 2:**
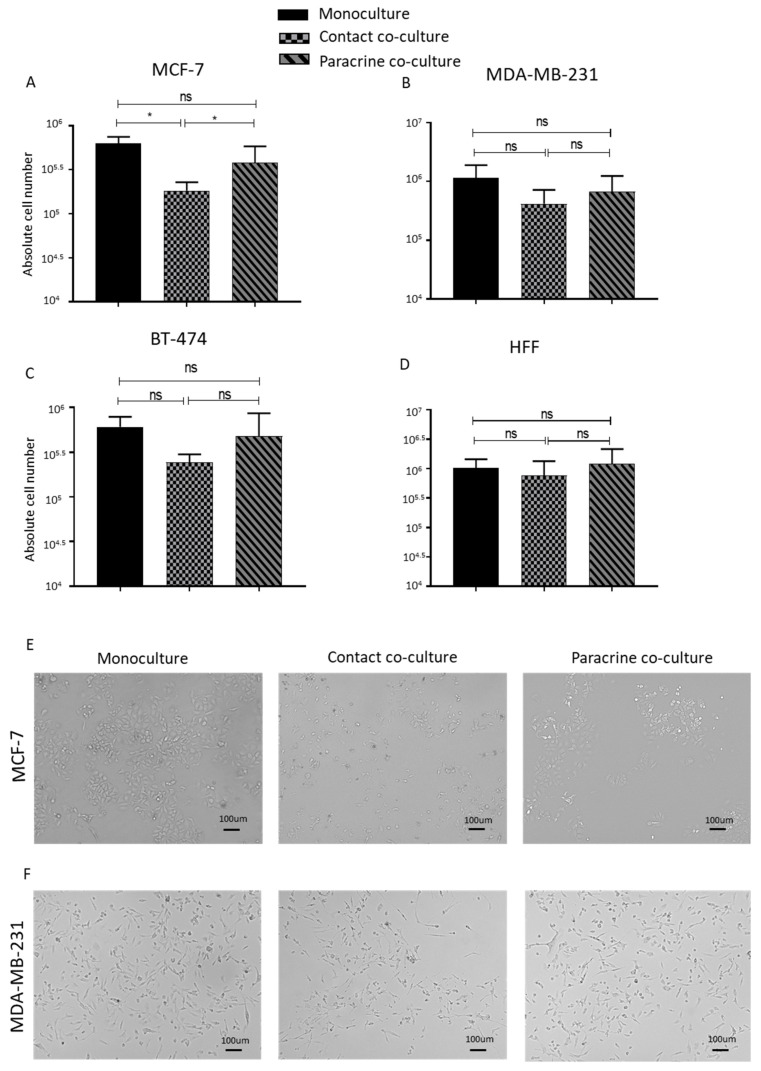
Contact and paracrine co-culture of lipoaspirate do not promote breast cancer cells’ proliferation. (**A**–**D**) Absolute cell count obtained using Neubauer counting chamber after 4 days of either contact or paracrine co-culture of lipoaspirates with MCF-7 (**A**), MDA-MB-231 (**B**), BT-474 (**C**), or human foreskin fibroblast (HFF) (**D**). Graphs are representative of the results obtained using lipoaspirate from 3 different donors. *p* value < 0.05 = *, ns = non-significant. (**E**,**F**) Bright field microscope images of contact and paracrine co-culture of MCF-7 and MDA-MB-231 cells with lipoaspirates.

The contact co-culture of lipoaspirates resulted in a significant decrease in the proliferation rate of the MCF-7 cells, while the MDA-MB 231 cells and BT-474 also showed a lower but non-significant proliferation rate on the contact culture (Figure 2A–C). Paracrine co-culture of lipoaspirate showed no effect on proliferation of breast cancer cell lines when compared to monocultured cells (Figure 2A–C). The human foreskin fibroblast (HFF) used as control demonstrated similar growth kinetics as monoculture upon both contact and paracrine co-culture with lipoaspirate (Figure 2D). We further confirmed our contact co-culture cell count results by fluorescent-based DNA measurement (Figure S1A,B). In addition, no enhancement in MCF-7 or MDA-MB-231 proliferation rate was observed upon co-culture with lipoaspirates obtained from cancer patients (Figure S1C,D). Titrating the proliferation pattern of MCF-7 in contact co-culture with lipoaspirates showed the largest drop in proliferation at day 3 post co-culture (Figure S1E). Microscopic images revealed a stressed morphology in the contact cultured MCF-7 and MDA-MB 231 cells (Figure 2E,F). We confirmed the viability of the lipoaspirates at the end of the co-culture experiments by isolating and culturing the adipose-derived stem cells following digestion of lipoaspirates with collagenase enzyme. Isolated ASCs demonstrated comparable morphology and growth kinetics to freshly isolated ASCs (data not shown). These results indicate that lipoaspirates do not promote the proliferation of breast cancer cells, rather suggesting that a contact-dependent proliferation inhibition is the most likely outcome.

### 2.2. Conditioned Mediums from Lipoaspirate Co-Culture Do Not Promote the Proliferation of Breast Cancer Cells in Culture

**Figure 3:**
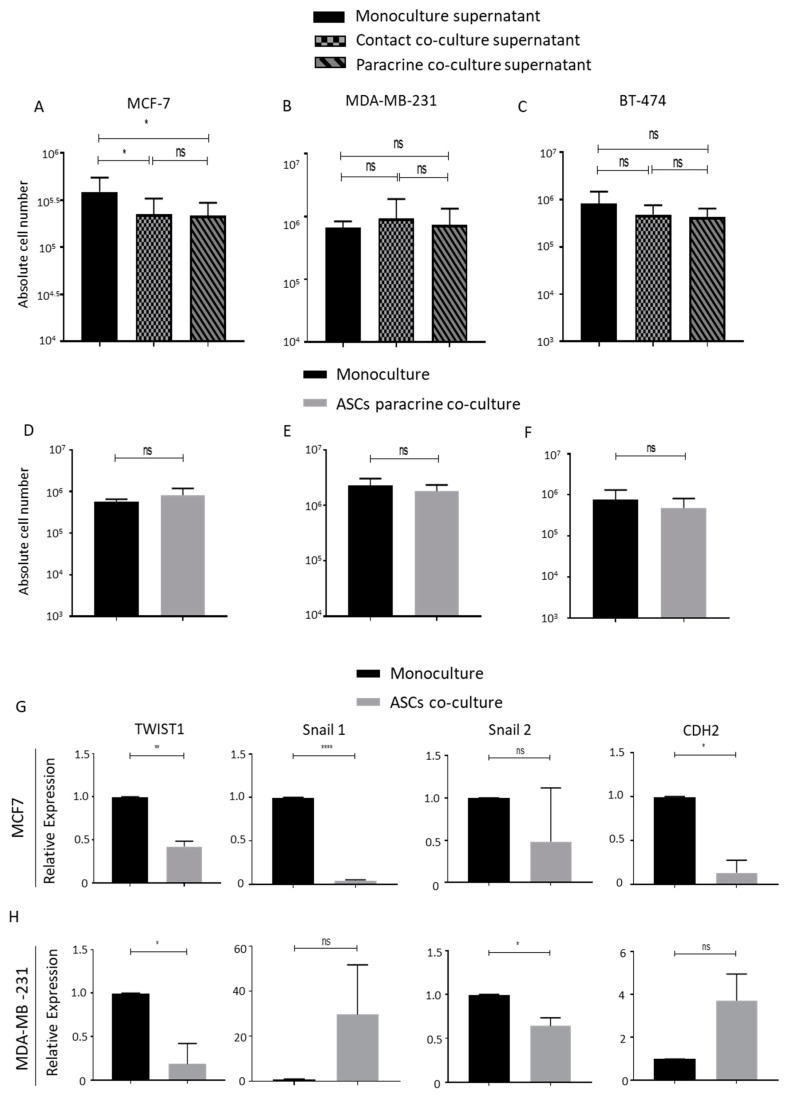
Transwell paracrine culture of ASCs does not promote breast cancer cells proliferation. (**A**–**C**) Absolute cell count obtained using Neubauer counting chamber after 4 days of culture of either MCF-7 (**A**), MDA-MB-231 (**B**) or BT-474 (**C**), in cell culture supernatant collected from either monocultured, contact-co-cultured or paracrine-cultured breast cancer cells with lipoaspirates. Graphs are representative of 3 independent experiments. (**D**–**F**) Representative graph of cell count of MCF-7 (**D**), MDA-MB-231 (**E**) and BT-474 (**F**) cell upon transwell co-culture with ASCs. ASCs from 3 different donors were employed. (**G**,**H**) Relative EMT genes expression of monocultured or co-cultured MCF-7 (**G**) and MDA-MB-231 (**H**) was analyzed by quantitative real-time PCR. *p* value < 0.05 = *, *p* < 0.01 = **, *p* < 0.0001 = ****, ns = non-significant.

We collected the cell culture supernatant from mono-cultured, contact-cultured and paracrine-cultured breast cancer cells lines with lipoaspirates. After filtration, we used this conditioned media to culture breast cancer cell lines. The cell counts after 5 days of culture indicated that conditioned media from both contact- and paracrine-co-cultured MCF-7 and lipoaspirates inhibited the proliferation of MCF-7 compared to cells cultured in supernatant from monoculture (Figure 3A). No change in the proliferation rates of MDA-MB-231 and BT-474 cells was observed in different conditioned mediums (Figure 3B,C, respectively). These results indicated that factors released by lipoaspirates selectively inhibit MCF-7 cells proliferation and lipoaspirate-conditioned medium does not promote breast cancer cells proliferation.

### 2.3. ASCs Contact Culture Inhibit the Proliferation of MDA-MB-231 Cells

Previous studies have reported conflicting results regarding the role of ASCs in promoting the proliferation and malignant transformation of breast cancer cells [2,14,15,16,22,25]. We isolated ASCs from the same donors from whom lipoaspirates were obtained and propagated them in vitro. Transwell co-cultures were performed by seeding breast cancer cells MCF-7, MDA-MB231 or BT-474 on the lower plastic surface, and ASCs were seeded in the transwell basket. Cell count analyses of breast cancer cells 5 days post co-culture revealed no differences in the growth kinetics of co-cultured breast cancer cells compared to mono-cultured cells (Figure 3D–F). EMT plays an important role in breast cancer metastasis. We analyzed the effects of the ASCs co-culture on the expression of EMT-related genes in breast cancer cell lines. Quantitative real-time PCR analyses of EMT genes *TWIST1, Snail1, Snail2* and *CDH2* in monocultured and ASCs co-cultured MCF-7 (Figure 3G) and MDA-MB-231 (Figure 3H) revealed that most of these EMT signature genes were downregulated in co-cultured breast cancer cells.

**Figure 4:**
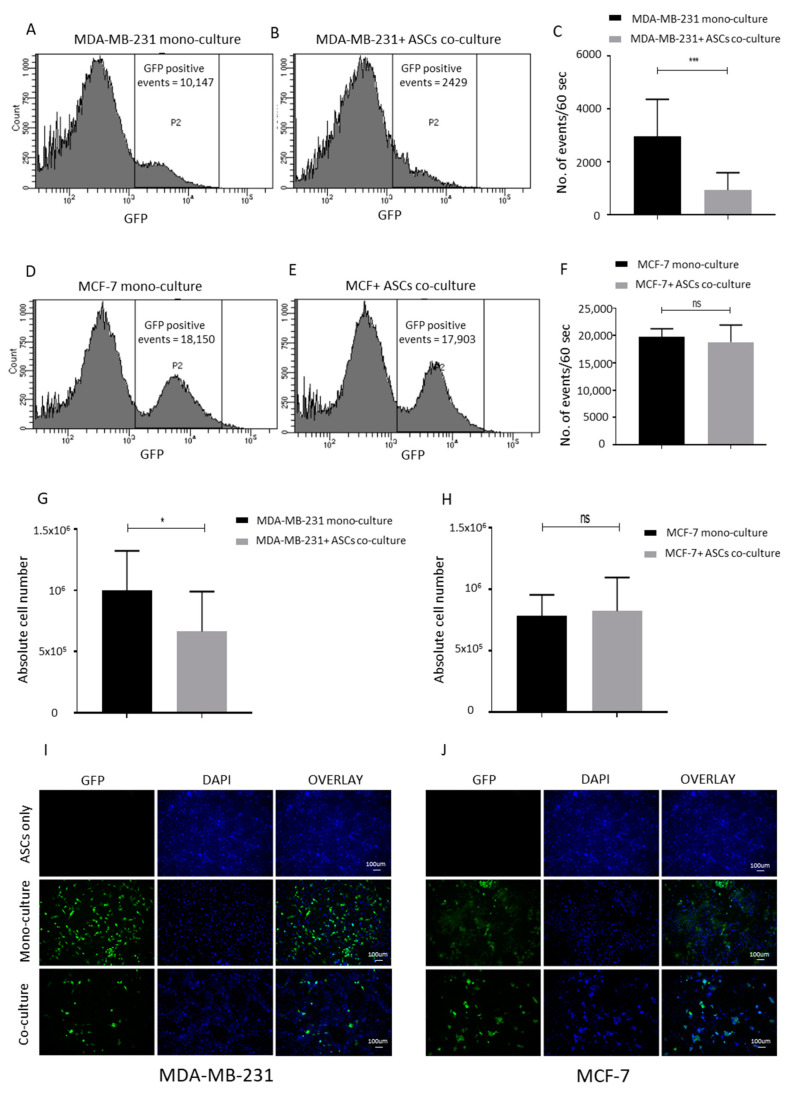
ASCs contact culture inhibits the proliferation of MDA-MB-231 cells. (**A**–**C**) Contact co-culture of MDA-MB-231 cells with ASCs. MDA-MB-231 expressing GFP were seeded either alone (**A**) or on confluent ASCs (**B**) for 4 days. The numbers of GFP-positive events per minute were counted 4 days post culture by using flow cytometry and plotted (**C**). (**D**–**F**) Contact co-culture of MCF-7 cells with ASCs. MCF-7 cells expressing GFP were seeded either alone (**D**) or on confluent ASCs (**E**) for 4 days. The numbers of GFP positive events per minute were counted 4 days post culture by using flow cytometry, and plotted (**F**). The graphs are representative of 3 independent experiments using ASCs from different donors. (**G**,**H**) Absolute number of cells counted by Neubauer chamber from the experimental settings (**A**,**B**) shown in (**G**) and from (**D**,**E**) as reflected in (**H**). (**I**,**J**) Fluorescent microscope images of MDA-MB-231/ASCs co-culture (**I**) and MCF-7/ASCs co-culture (**J**) are shown. GFP-positive cells are green while nuclear stain DAPI stain blue. *p* value < 0.05 = *, *p* < 0.001 = ***, ns = non-significant.

Since our lipoaspirates contact co-culture studies showed inhibitory effects on the proliferation of MCF-7 cells, we planned a contact co-culture of ASCs with breast cancer cells MCF-7 and MDA-MB-231. GFP-expressing MCF-7 and MDA-MB-231 cell lines were generated and sorted (Figure S2A,B). ASCs were grown to full confluency and also mixed with the monocultured GFP-expressing breast cancer cells as shown in (Figure S2C) to achieve a consistency in downstream analyses. A FACS event count for 60 s at a constant flow rate revealed a significantly lower green events count upon the co-culturing of ASCs with GFP-MDA-MB-231 cells (Figure 4A–C). No significant differences were observed in the events count of monocultured and co-cultured GFP-MCF-7 cells (Figure 4D–F). The FACS count results were further confirmed by manually counting the total number of the cells (Figure 4G,H) and by the visualization of green cells using fluorescent microscopy (Figure 4I,J). We conclude that neither the paracrine nor the contact co-culture of ASCs promote the proliferation of ASCs; rather, a direct cell–cell contact of ASCs with MDA-MB-231 cells reduces the MDA-MB-231 cells’ proliferation rate.

### 2.4. Lipoaspirate Contact Co-Culture Inhibit Cell Cycle in MCF-7 Cells

**Figure 5:**
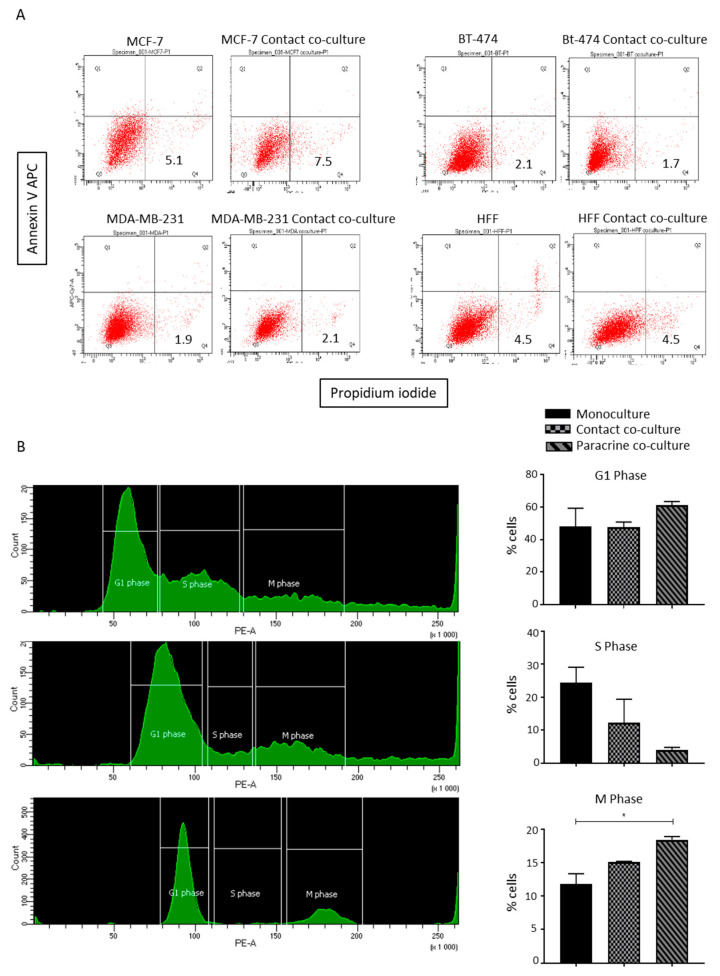
Lipoaspirate contact culture results in cell cycle arrest in MCF-7 cells. (**A**) Effect of lipo-aspirate contact co-culture on breast cancer cell survival. AnnexinV/Propidium Iodide staining was employed to estimate the cell death of breast cancer cells upon co-culture. (**B**) Cell cycle analyses of paracrine and contact-co-cultured MCF-7 cells with lipo-aspirates. Graphs show the percentages of cells in different phases of cells upon different culture conditions. *p* value < 0.05 = *.

To understand the possible underlying mechanism involved in the lipoaspirate contact co-culture-mediated reduction in MCF-7 proliferation, we first analyzed a possible induction of apoptotic cell death in co-cultured breast cancer cells. Annexin/PI staining of co-cultured cells showed no notable changes in annexin/PI-positive cell percentages (Figure 5A).

Next, we analyzed the distributions of cells in different phases of the cell cycle by trypsanizing and staining the fixed cells with propidium iodide. FACS analyses of the DNA content revealed that upon the contact co-culture of MCF-7 cells with lipoaspirates, the majority of MCF-7 cells were arrested in either the G1 or M phase, and a very small percentage of the cells were in the S phase as compared to the monocultured cells (Figure 5B, left and right panel). The paracrine co-cultured cells showed a comparable distribution of cells to monocultured cells. Of note, paracrine- and contact-co-cultured MDA-MB-231 cells showed no differences in the distribution of the cells in different phases of the cell cycle compared to monocultured cells (data not shown).

### 2.5. Lipoaspirate Contact Co-Culture and Supernatant Activate Retinoblastoma Protein (RB) Mediated Cell Cycle Arrest in MCF-7 Cells

**Figure 6:**
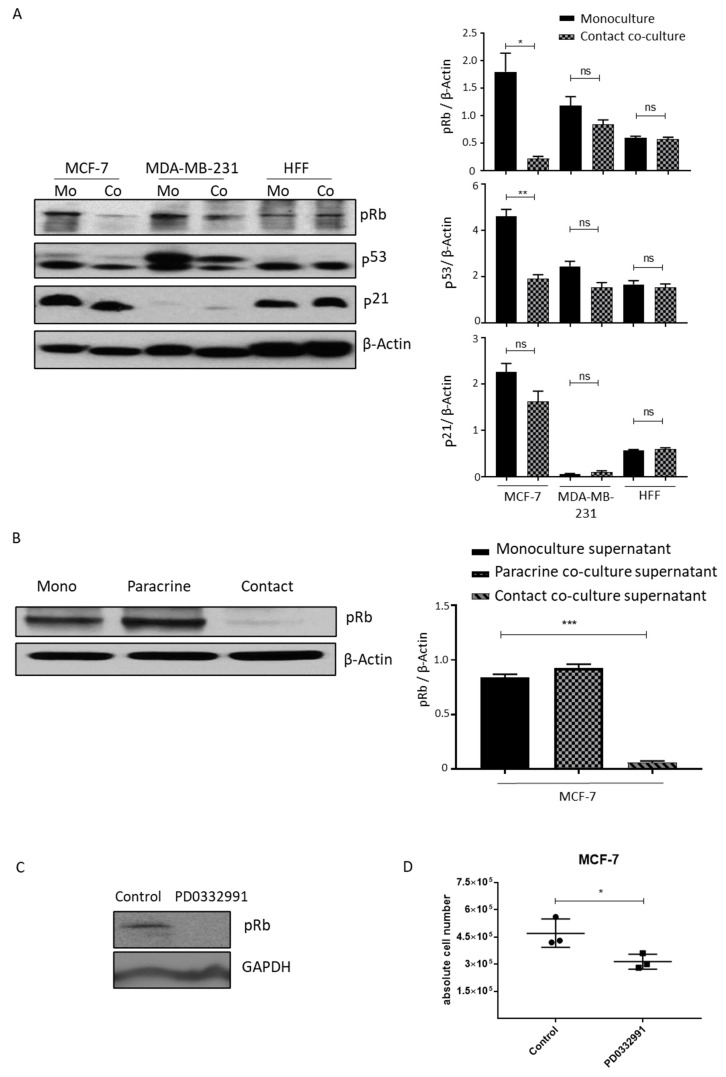
Contact lipoaspirate culture inhibits MCF-7 proliferation via the retinoblastoma-mediated pathway. (**A**) Cell lysates from MCF-7, MDA-MB-231 or HFF contact-co-cultured with lipo-aspirates were collected in Ripa lysate buffer. The phosphorylation of retinoblastoma protein (Rb) and the expression of p53 and p21 were analyzed by western blotting using specific antibodies. β-Actin was employed as the loading control. Fold changes in densitometric band intensities, acquired by image J and normalized to β-Actin, were compared and plotted. (**B**) Cell lysates from MCF-7 cells cultured in media from either monocultured, paracrine-cultured or contact-cultured MCF-cells with lipo-aspirates were collected in Ripa buffer and blotted for phosphorylated retinoblastoma protein (Rb). β-Actin was employed as the loading control. (**C**,**D**) MCF-7 cells were incubated with 10µM PD0332991 and cell lysates were blotted for Rb protein phosphorylation (**C**) and counted at day 4 post seeding (**D**). *p* value < 0.05 = *, *p* < 0.01 = **, *p* < 0.001 = ***, ns = non-significant.

Mitogenic stimulation results in cyclin-dependent, kinase-mediated phosphorylation of the RB protein, thus inhibiting their growth inhibitory functions and allowing the cells to enter the S phase of the cell cycle [26]. We analyzed the expression of cell cycle regulators in MCF-7 and MDA-MB-231 cells contact-co-cultured with lipoaspirates. Consistent with the lower proliferation rate and cell cycle arrest observed in co-cultured MCF-7 cells, we observed a significant decrease in the phosphorylation of RB protein, reflecting enhanced cell cycle inhibitory function of RB protein (Figure 6A, left and right panel). No significant effect on RB phosphorylation was observed in lipoaspirate contact-co-cultured MDA-MB-231 or control HFF cells (Figure 6A, left and right panel). We observed a decrease in the expression of the tumor suppressor p53 but no changes in p21 protein levels in MCF-7 and MDA-MB-231 cells, indicating a possible compensatory regulation of p53 expression (Figure 6A, left and right panel). To confirm the possible role of RB protein activity in the reduced proliferation of co-cultured MCF-7 cells, we cultured MCF-7 cells in the supernatant collected from the lipoaspirate paracrine and contact culture experiment. Western blot analyses of these MCF-7 cells revealed that the supernatant from contact culture experiments inhibits RB protein phosphorylation (Figure 6B, left and right panel). The inhibition of RB protein phosphorylation in MCF7 cells using CDK 4/6 inhibitor PD0332991 resulted in reduced proliferation (Figure 6C,D). We conclude that lipoaspirate contact culture inhibits MCF-7 proliferation via RB protein.

## 3. Discussion

AFT has been widely adapted for breast reconstruction procedures given its autologous nature and regenerative properties. However, the oncological safety of AFT has been hotly debated, with opposing basic science and clinical literature findings that have polarized plastic surgeons’ and oncologists’ opinions on the cost–benefit tradeoffs of AFT. Despite the large number of studies surrounding the topic, the field still lacks a clear direction due to conflicting reports from the variable choices of in vitro and in vivo study models. For example, in vitro studies utilized ASCs, in vitro differentiated adipocytes or floating adipocytes as the co-culture interaction partner of breast cancer cells, and reported the tumorigenic behavior of the breast cancer cells [14,16,20,22]. These interactive studies may not reflect the actual clinical scenario of AFT, where lipoaspirates are injected into tumor beds that may or may not have remnant active tumor cells. Lipoaspirates represent a smaller unit of fat tissue that still comprise a cellular milieu, including adipocytes, ASCs, preadipocytes, pericytes, endothelial cells and hematopoietic lineage cells [27]. The in vitro propagation of cells outside their natural microenvironment alters their behavior. Gebremeskel et al. showed the higher expression of tumorigenic genes, such as Arg-1, TGF-β and VEGF, in cultured ASCs as compared to fat graft [28]. Thus, in vitro studies involving ASCs, in vitro-differentiated adipocytes or floating adipocytes may not be the true reflection of the interaction of these cells with breast cancer cells in a clinical setting. To overcome these possible technical shortcomings and simulate a more accurate clinical scenario, we carried out a cell–cell contact and a paracrine interaction study between fresh lipoaspirates and breast cancer cell lines. To our knowledge, this is the first attempt to study the contact-dependent interaction between lipoaspirates and breast cancer cells. We performed these studies in a closed flask culture system to prevent the exposure of the floating lipoaspirates to the air interface during the culture, which can result in possible cell death and the release of pro-inflammatory cytokines into the culture medium.

Employing our lipoaspirate co-culture system, we observed a significant decrease in the proliferation rate of MCF-7 cells in both the paracrine and contact co-culture settings. The other tested breast cancer cell lines MDA-MB-231 and BT-474 showed no difference in the proliferation pattern compared to the mono-cultured cells. Similarly, our control cell line HFF showed no notable effect of co-culturing with lipoaspirates on proliferation. These results indicate that lipoaspirate selectively inhibits MCF-7 cells’ proliferation in vitro, and likely has no impact on normal healthy cells. We have previously shown that lipofilling following MCF-7 cells engraftment resulted in significantly lower tumor volume and mass, and showed significantly lower Ki-67 proliferation index [23]. A separate study showed that free fatty acids released by subcutaneous fat inhibit the proliferation of MCF-7 as a possible mechanism [29]. In line with our in vitro results showing no changes in MDA-MB-231 cells’ proliferation pattern in co-culture, fat grafting studies in mice showed no significant changes in the MDA-MB-231 tumor volume [22,25]. On the other hand, two independent studies using a transwell paracrine co-culture system showed an increase in the MCF-7 cells’ proliferation upon incubation with lipoaspirate [20,21]. One possibility for this contrary observation could be the potential interference resulting from the damage induced by the exposure of the lipoaspirate to the air interface, which results in rapid phenotype loss due to hypoxia and inflammation [30,31]. Noting this potential problem with adipocyte culture, a recent study has shown an ‘under membrane’ transwell alternative culture technique, which maintains the identity and function of adipocytes. This technique can be used as a potential technical resource for performing paracrine interaction studies [32]. Using our flask culture, we also avoided the potential interference of hypoxia-induced inflammation in our culture conditions. In addition, the supernatant from the lipoaspirate co-culture showed an inhibitory effect on the proliferation of MCF-7 cells.

Our result from the paracrine transwell co-culture of ASCs with breast cancer cells demonstrated no significant change in the proliferation of MCF-7, MDA-MB-231 or BT-474 cells. In these studies, we used ASCs isolated from the same lipoaspirates donors to comprehensively compare the effects of different cell processing techniques, from the same source, on tumorigenic outcomes. Although ASCs alone are not currently employed as a tool for reconstruction procedures, they constitute a major fraction of the adipose tissue stromal vesicular fraction (SVF). Moreover, in some AFT protocols, SVF-enriched lipoaspirates are used to enhance the graft volume retention [33]. One clinical study found no significant difference in cancer recurrence in patients who received SVF-enriched lipoaspirates for breast reconstruction as compared to the control group [33]. Similarly, co-culture with ASCs showed no significant effect on the proliferation of MCF-7 or MDA-MB-231 cells [2]. Contrarily, Ritter et al. reported an increase in the proliferation of the MCF-7 cells upon co-culture with ASCs [34]. A possible explanation for these differing observations could be the use of different ASCs ratios in the co-culture experiments. We used a 1:6 MCF-7 to ASCs ratio in our co-culture experiment compared to the 5:1 ratio used by Ritter et al. The choice of the 1:6 MCF-7 to ASCs ratio in our study was made considering the clinical scenario of AFT, which is performed after confirmation of no active cancer. Therefore, very few remnant cancer cells are expected, even if some survive radio- and chemotherapy. Supporting this reasoning, Ryu et al. have shown that high-density cultured ASCs even suppress the growth of MCF-7 cells [35]. Our cell–cell contact culture also showed no increase in the proliferation of MCF-7 cells. Intriguingly, we instead observed a significant decrease in the proliferation rate of MDA-MB-231 cells. Previous studies reported a similar inhibitory effect of ASCs contact culture on the proliferation of MDA-MB-231 cells, most likely through a (TNF)-α-related apoptosis-inducing ligand (TRAIL)-mediated interaction [34,36]. Finally, our results showed retinoblastoma protein mediated cell cycle arrest as a possible mechanism involved in the lipoaspirate co-culture-dependent inhibition of MCF-7 cells proliferation. Interestingly, we observed a downregulation of p53 protein expression in MCF-7 cells, which most likely is a compensatory mechanism to balance Rb protein activity [37].

## 4. Materials and Methods

### 4.1. Donor Specification

Adipose tissue was collected from 5 female donors in the age range 39 ± 13 and BMI range 27 ± 4 undergoing elective plastic surgery procedures at the University of Pittsburgh Medical Center (UPMC). The procedure of tissue collection was approved by the University of Pittsburgh Institutional Review Board (IRB No. 0511186). Fat tissue aspirates were collected in 20 mL syringes and centrifuged at 3000 rpm for 5 min. The upper oil layer and lower aqueous layer were discarded, and the middle layer was washed with PBS and used in co-culture experiments.

### 4.2. Isolation of Human Adipose-Derived Stem Cells

Human subcutaneous white adipose tissue (sWAT) samples were harvested from donors undergoing routine abdominoplasty at the Department of Plastic Surgery, University of Pittsburgh. The tissue collection methods were approved by the Institutional Review Board (IRB No. 0511186). All sWAT samples were obtained from the lower abdomen. Adipose tissue biopsies after surgery procedures were transferred to the lab in sterile containers before processing in a laminar flow sterile work bench class II. Tissue was rinsed 3 times with PBS (Sigma, St. Louis, MO, USA), followed by the removal of fibrous material and blood vessels by dissection.

The tissue was cut into pieces (~1–2 mg) and digested in digestion buffer (PBS) containing 200 U/mL collagenase (CLS Type II, Worthington Biochemical Corp., Lakewood, NJ, USA) and 2% *w/v* BSA (Sigma, St. Louis, MO, USA) under stirring for 60 min at 37 °C and 450 rpm; 1 g adipose tissue/3 mL digestion buffer. The dispersed tissue was centrifuged for 10 min at 200 RCF at room temperature. The floating adipocytes were aspirated, and the sedimented stromal-vascular fraction (SVF) was suspended in erythrocyte lysis buffer (0.155 M NH4CI, 5.7 mM K2HPO4, 0.1 mM EDTA, pH 7.3) and incubated for 10 min at room temperature.

To remove tissue debris, the cell suspension was filtered through a nylon mesh (pore size 100 μm, BD, USA). After another centrifugation step (10 min at 200 RCF), the pelleted SVF was suspended in DMEM/F12 medium (Sigma, St. Louis, MO, USA), supplemented with 10% FBS (Sigma, St. Louis, MO, USA), and filtered through a 40 μm mesh to remove residual cell aggregates. SVF cells were inoculated into cell culture flasks at a density of 30,000/cm^2^. The attached cell population at one-week post-culture was referred to as the adipose-derived stromal cell (ASC) fraction and used for further studies. The ASC cell population contained an enriched population of adipose stem/progenitor cells.

### 4.3. Culture of ASCs

Upon reaching 70% confluence in the T175 flask (Thermo Fisher Scientific, Waltham, MA, USA), the ASCs were washed with phosphate buffered saline (PBS, Thermo Fisher Scientific, Waltham, MA, USA) and trypsinized using 3 mL 0.5% trypsin-EDTA 1x (Sigma, St. Louis, MO, USA). Trypsin was neutralized using 7 mL DMEM/F12 medium, 10% FBS medium and 50 µg/mL Gentamicin (Invitrogen, Carlsbad, CA, USA), and removed by centrifugation at 300 RCF for 5 min. The cells were again seeded at a density of 5000–7000 cells/cm^2^ in DMEM/F12 medium plus 10% FBS, and maintained at 37 °C with 5% CO_2_. ASCs were passaged by seeding at 5000–7000 cells/cm^2^, the medium was changed every third day and the cells were grown to 70% confluence before splitting. ASCs cultivated to passage 3–4 were used in this study.

### 4.4. Culture of Breast Cancer Cell lines

BT-474, MDA-MB-231 and MCF-7 cell lines were purchased from American Type Culture Collection (ATCC, Manassas, VA, USA), and cultured in DMEM medium (Sigma, St. Louis, MO, USA) supplemented with 10% FCS and antibiotics. Cells were cultured at 37 °C and 5% CO_2_.

### 4.5. Culture of Human Foreskin Fibroblasts (HFF)

Human foreskin fibroblasts were obtained from ATCC and cultivated using methods described by Cavinato et al. [38]. Briefly, 5000 cells/cm^2^ were seeded in T25 flask in DMEM (Sigma, St. Louis, MO, USA) medium supplemented with 10% FCS.

### 4.6. Inverted Flask Culture: Contact Co-Culture

MCF-7, MDA-MB 231, BT-474 or HFF 1 × 10^5^ cells were seeded in vented T12.5 flasks (Corning, NY, USA) in 3 mL DMEM, 10% FCS medium and cultured overnight at 37 °C and 5% CO_2_. After 24 h, 1 mL lipoaspirate was added in the flask and the flasks were filled completely with medium. Following lipoaspirate addition, the flasks were cultured with the bottom side seeded with cultured breast cancer cells. The flasks were inverted such that the cells faced down, as shown in Figure 1D. Since lipoaspirate is buoyant and tends to float on the surface of the medium, this arrangement allowed the lipoaspirate to float to the top of the inverted flask, thus maintaining contact with the seeded breast cancer cells. Co-cultures were performed for 4 days without performing any media change unless indicated, and the proliferation outcome was recorded by either cell count using Neubauer chamber or fluorescent DNA quantification-based assay Cy-QUANT (Thermo Fisher Scientific, Waltham, MA, USA).

### 4.7. Conventional Flask Culture: Paracrine Co-Culture

MCF-7, MDA-MB 231, BT-474 or HFF 1 × 10^5^ cells were seeded in T12.5 flasks (Corning, NY, USA) in 3 mL DMEM, 10% FCS medium and cultured overnight at 37 °C and 5% CO_2_. After 24 h, 1 mL lipoaspirate was added in the flask and flasks were filled completely with medium. Flasks were cultured in conventional culture position as shown in Figure 1C. This arrangement allowed the lipoaspirate to float to the top and created a paracrine co-culture environment where both cancer cells and lipoaspirates were cultured in the same flask without being in contact. Following 4 days of co-culture, the cells were counted using a Neubauer chamber.

### 4.8. Transwell Co-Culture Experiments

A Transwell system (0.4 µm pore size, Polyester (PET) membrane; Corning, Corning, NY, USA) was employed. Either 5 × 10^4^ MCF-7 or MDA-MB231 or BT-474 were cultured in the bottom surface of a 6-well plate. 3 × 10^5^ Human ASCs were cultured in the upper transwell basket. The co-culture was performed for 5 days followed by a cell count using a Neubauer chamber. Wells without ASCs in the transwell were counted as control and compared with co-cultured cell counts.

### 4.9. Generation of GFP-Expressing Breast Cancer Cell Lines

GFP-expressing MCF-7 and MDA-MB-231 cell lines were generated by transfecting the cells with p-Lenti-CMV-MCS-GFP-SV-puro plasmid (addgene Plasmid # 73582). Cells were grown to 70% confluency in 6-well plates and transfected with 2ug plasmid DNA using lipofectamine 2000 (Thermo Fisher Scientific, Waltham, MA, USA) following manufacturer’s protocol. After 48 h of transfection, cells were selected with puromycin for 1 week. GFP-positive cells were sorted by FACS and used in co-culture experiments (Figure S2A,B).

### 4.10. ASCs Breast Cancer Cells Contact Co-Culture

ASCs were grown to full confluency in 6-well plates at an initial seeding density of 3 × 10^5^ cells per well. 5 × 10^4^ MCF-7 or MDA-MB-231 cells expressing GFP were seeded over the confluent ASCs layer and co-cultures were performed for 5 days (Figure S2C). At the end of the co-culture duration, the cells were either fixed for fluorescent microscopy or trypsinized for counting by FACS or Neubauer chamber. As a control, monocultured confluent ASCs and 5 × 10^4^ MCF-7 or MDA-MB-231 cells cultured for 5 days were trypsinized and pooled together as shown in (Figure S2C) for FACS or Neubauer counting experiments. Monocultured wells were fixed for fluorescent microscopy.

### 4.11. GFP Positive Cell Count by FACS

Following five days of co-culture, ASCs and MCF-7 or MDA-MB-231 cells were trypsinized, washed and resuspended in 500 μL PBS/1%BSA buffer. Monocultured ASCs or MCF-7 or MDA-MB-231 wells were trypsinized, pooled, washed and resuspended in 500 μL PBS/1%BSA buffer. Cells were subjected to flow cytometer (FACS Fortessa, BD, Franklin Lakes, NJ, USA) at a medium aspiration rate and the recording was performed after 20 s of initial aspiration for 1 min. The number of GFP positive events per minute was counted using FACS Diva software (BD, Franklin Lakes, NJ, USA).

### 4.12. CyQUANT Cell Proliferation Assay

Co-cultured cell proliferation was monitored using CyQUANT proliferation assay (Thermo Fisher Scientific, Waltham, MA, USA) following the manufacturer’s protocol. Briefly, following co-culture, the supernatant was removed, and cells were trypsinized and pelleted. The cell pellet was lysed by adding the lysis buffer containing the dye (provided in the kit). Fluorescence was measured in a microplate reader (Tecan, Switzerland) with excitation at 485 nm and emission detection at 530 nm.

### 4.13. Fluorescent Microscopy

Cells were fixed using 4% paraformaldehyde for 30 min. Nucleus was stained with DAPI and images were taken using fluorescent microscope and software (Keyence, Itasca, IL, USA).

### 4.14. Annexin/Propidium Iodide Live Dead staining

Annexin/PI live/dead staining was performed using an APC Annexin V Apoptosis Detection Kit with PI (BioLegend, San Diego, CA, USA) following the manufacturer’s protocol. Stained cells were analyzed by FACS (FACS Fortessa, BD, Franklin Lakes, NJ, USA) and data were analyzed using FACS Diva software.

### 4.15. Cell Cycle Analyses

The cell cycle was analyzed by fixing the trypsinized cell with 70% ice cold ethanol and staining the cells with propidium iodide (Sigma, St. Louis, MO, USA). Cells were analyzed by FACS and data were acquired and analyzed by FACS Diva software.

### 4.16. Western Blotting

Western blotting was performed as published earlier [39]. Blots were developed using primary Phospho-Rb (Ser807/811), p53, p21, beta actin and HRP-linked species-specific secondary antibodies (Cell Signaling Technology, Danvers, MA, USA).

### 4.17. Quantitative Real-Time Polymerase Chain Reaction (PCR)

RNA was extracted from cells using the RNeasy Mini Kit (Qiagen, MD, USA) following the manufacturer’s instruction. RNA was reverse transcribed using High Capacity cDNA Reverse Transcription Kit (Applied Biosystems, Foster City, CA, USA) according to manufacturer’s protocol. Using cDNA as a template and gene-specific primers (Sigma, St. Louis, MO, USA), the expression of Twist1, Snail1, Snail 2 and CDH2 was quantified by quantitative real time PCR using the QuantStudio 3 Real-time PCR (ThermoFisher Scientific, Waltham, MA, USA). Data for each gene transcript were normalized by calculating the difference (∆Ct) from the Ct-housekeeping and Ct-Target genes. The relative increase or decrease in expression was calculated by comparing the reference gene with the target gene (∆∆Ct) and using the formula for relative expression (=2∆∆Ct).

### 4.18. Statistical Analyses

Data are reported as the mean ± standard error of mean. We assessed the subject variability of the measured outcome. Student’s T test and an Analysis of Variance (ANOVA) test were performed where applicable using GraphPad Prism software. Error bars are represented as the mean ± SEM.

### 4.19. Ethics Approval and Consent to Participate

Adipose tissue from donors undergoing elective plastic surgery procedures at the University of Pittsburgh Medical Center (UPMC) were employed for this study. The procedure of tissue collection was approved by the institutional review board (IRB No.0511186).

## 5. Conclusions

To summarize, our in vitro contact and paracrine co-culture results support the oncological safety of AFT. Future studies should utilize in vivo breast cancer cell titration studies in the presence of lipoaspirates in mice humanized for immune system. Other lines of inquiry might include broader and more rigorous analyses of tumor recurrence among breast cancer patients who received AFT for breast reconstruction. Such data will help to develop conclusive guidelines for the use of AFT for breast reconstruction post-oncological treatment.

List of abbreviations: adipose-derived stem cells (ASCs); autologous fat grafting (AFT); stromal vascular fraction (SVF); retinoblastoma (Rb).

## Supplementary Materials

Supplementary materials can be found at https://www.mdpi.com/1422-0067/21/23/9171/s1. All data generated or analyzed during this study are included in this published article [and its supplementary information files].
