# Supplementary material for: The Impact of Human Lipoaspirate and Adipose Tissue-Derived Stem Cells Contact Culture on Breast Cancer Cells: Implications in Breast Reconstruction
Source: Int J Mol Sci. 2020 Dec 1;21(23):9171. doi: 10.3390/ijms21239171 (PMC7731376; doi:10.3390/ijms21239171)
Supplement: Supplementary file 1 [file ijms-21-09171-s001.pdf]

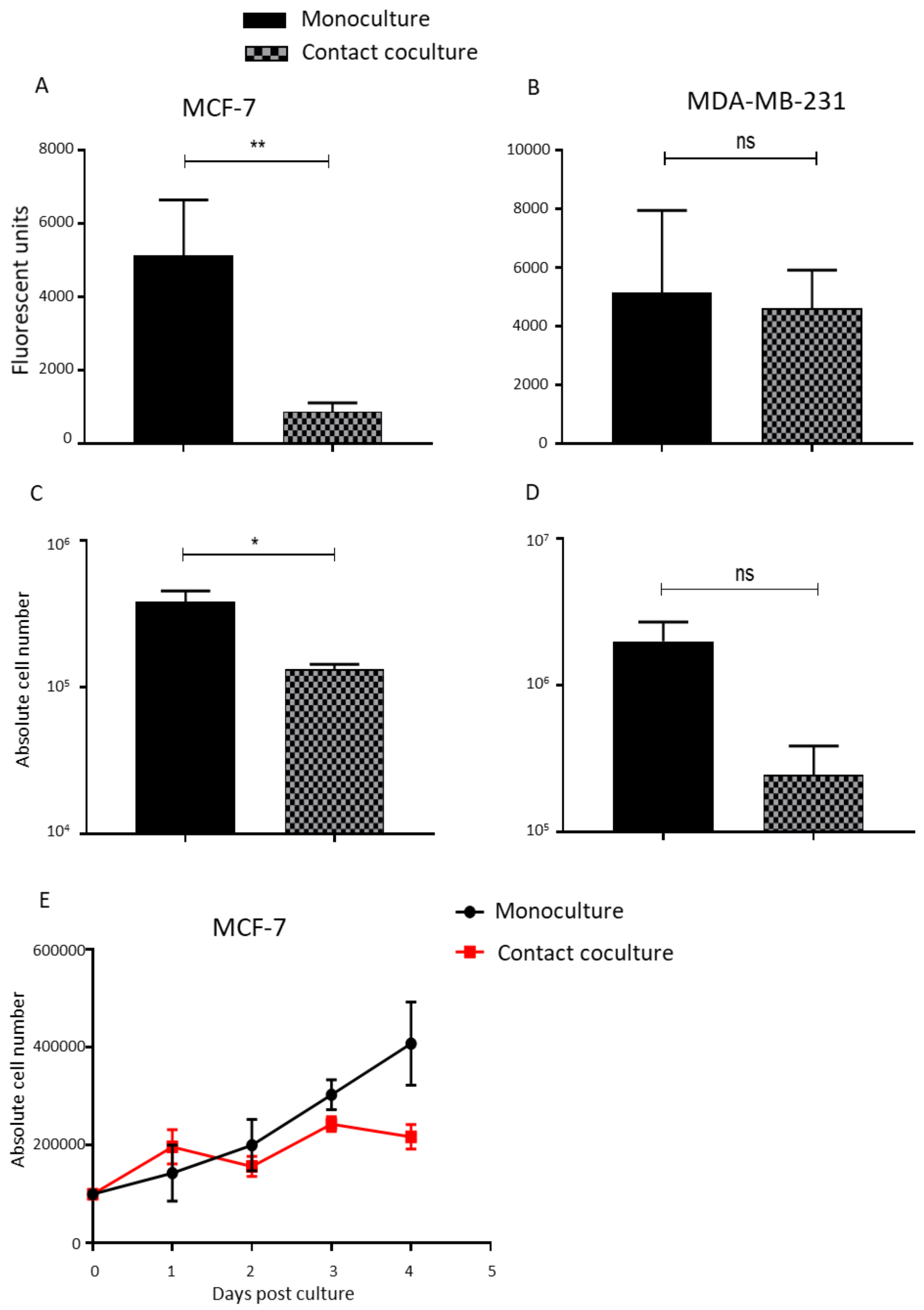

Figure S1. (A and B) Effect of contact co-culture of MCF-7 (A) or MDA-MB-231 (B) cells with lipo-aspirates was monitored employing Cy-Quant® cell proliferation assay. (C and D) Absolute cell count obtained using Neubauer counting chamber after 4 days of contact co-culture of lipoaspirates with MCF-7 (C) or MDA-MB-231 (D). Graphs are representative of results obtained using lipo-aspirate from 2 different cancer patients. P value < 0.05 = *, ns= non-significant. (E) Proliferation pattern of monocultured or lipoaspirate co-cultured MCF-7 cells was monitored for 4 days using Neubauer counting chamber.

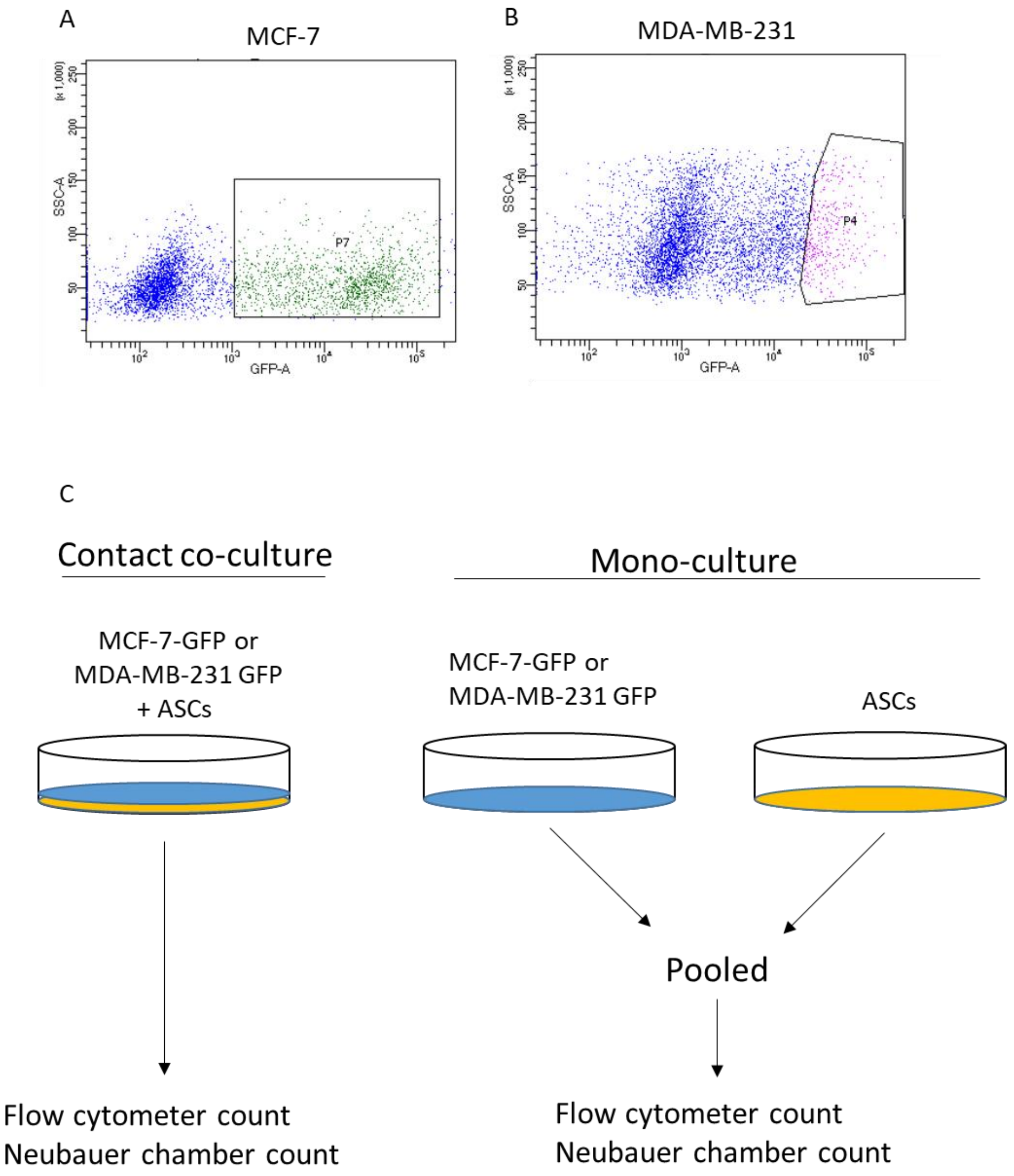

Figure S2. (A and B) MCF-7 (A) or MDA-MB-231 (B) cells were tranduced by lentiviral particles expressing GFP and sorted based on GFP positivity. (C) Schematic presentation of GFP expressing MCF-7 or MDA-MB-231 cancer cells contact co-culture with ASCs.
